# Epigenetic Mechanisms in Memory and Cognitive Decline Associated with Aging and Alzheimer’s Disease

**DOI:** 10.3390/ijms222212280

**Published:** 2021-11-13

**Authors:** Sabyasachi Maity, Kayla Farrell, Shaghayegh Navabpour, Sareesh Naduvil Narayanan, Timothy J. Jarome

**Affiliations:** 1 Department of Physiology, Neuroscience and Behavioral Sciences, St. George’s University School of Medicine, Grenada, West Indies; SMaity@sgu.edu; 2 Department of Animal and Poultry Sciences, Virginia Polytechnic Institute and State University, Blacksburg, VA 24061, USA; kaylac15@vt.edu; 3 Translational Biology, Medicine and Health, Fralin Biomedical Research Institute, Roanoke, VA 24016, USA; navabpour@vt.edu; 4 Department of Physiology, Ras Al Khaimah College of Medical Sciences, Ras Al Khaimah Medical & Health Sciences University, Ras Al Khaimah P.O. Box 11172, United Arab Emirates; sareesh@rakmhsu.ac.ae; 5 School of Neuroscience, Virginia Polytechnic Institute and State University, Blacksburg, VA 24061, USA

**Keywords:** epigenetics, DNA, histone, hippocampus, memory, neurodegeneration

## Abstract

Epigenetic mechanisms, which include DNA methylation, a variety of post-translational modifications of histone proteins (acetylation, phosphorylation, methylation, ubiquitination, sumoylation, serotonylation, dopaminylation), chromatin remodeling enzymes, and long non-coding RNAs, are robust regulators of activity-dependent changes in gene transcription. In the brain, many of these epigenetic modifications have been widely implicated in synaptic plasticity and memory formation. Dysregulation of epigenetic mechanisms has been reported in the aged brain and is associated with or contributes to memory decline across the lifespan. Furthermore, alterations in the epigenome have been reported in neurodegenerative disorders, including Alzheimer’s disease. Here, we review the diverse types of epigenetic modifications and their role in activity- and learning-dependent synaptic plasticity. We then discuss how these mechanisms become dysregulated across the lifespan and contribute to memory loss with age and in Alzheimer’s disease. Collectively, the evidence reviewed here strongly supports a role for diverse epigenetic mechanisms in memory formation, aging, and neurodegeneration in the brain.

## 1. Introduction

The concept of epigenetics was first introduced by Waddington to explain how different phenotypes can arise without changes in genotype [1]. All somatic cells from multicellular organisms, like humans, have an identical genome, but during development, cells are still able to differentiate into structurally and functionally different cell types. The underlying mechanisms for how cells with identical genomes are able to exhibit such a structural and functional difference has been a long-standing question, but the concept of epigenetics provides an explanation. Waddington proposed that the functional differences in genetically identical cells are most likely due to mechanisms occurring above the level of gene coding by the DNA [1]. We now know that epigenetic mechanisms are responsible for controlling gene transcription of individual cells through changes in chromatin structure as well as changes to DNA and chromatin accessibility. This mechanism creates differential gene expression between cells, though the underlying genome remains identical. As many definitions exist [2], in this review we define epigenetics as changes to chromatin structure and DNA and chromatin accessibility.

Outside of its role in cell differentiation during development, there is mounting evidence suggesting that epigenetic mechanisms play a significant role in transcriptional control during memory formation in the brain [3,4]. Importantly, age-related changes in these mechanisms have been strongly implicated with memory loss across the lifespan [5]. Additionally, dysregulation of epigenetic mechanisms has been widely implicated in various disease states, ranging from cancers to numerous neurological, psychiatric, and neurodegenerative disorders [6]. In this review, we will discuss the different types of epigenetic modifications that have been identified and how they contribute to synaptic plasticity and memory formation in the brain. Next, we will review findings indicating that these mechanisms become dysregulated across the lifespan, leading to age-associated memory loss, a risk factor for the development of neurodegenerative disorders such as Alzheimer’s disease. Finally, we conclude by reviewing evidence indicating that the dysregulation of epigenetic mechanisms is associated with, and might contribute to, memory loss associated with Alzheimer’s disease.

## 2. Types of Epigenetic Mechanisms

**Figure 1:**
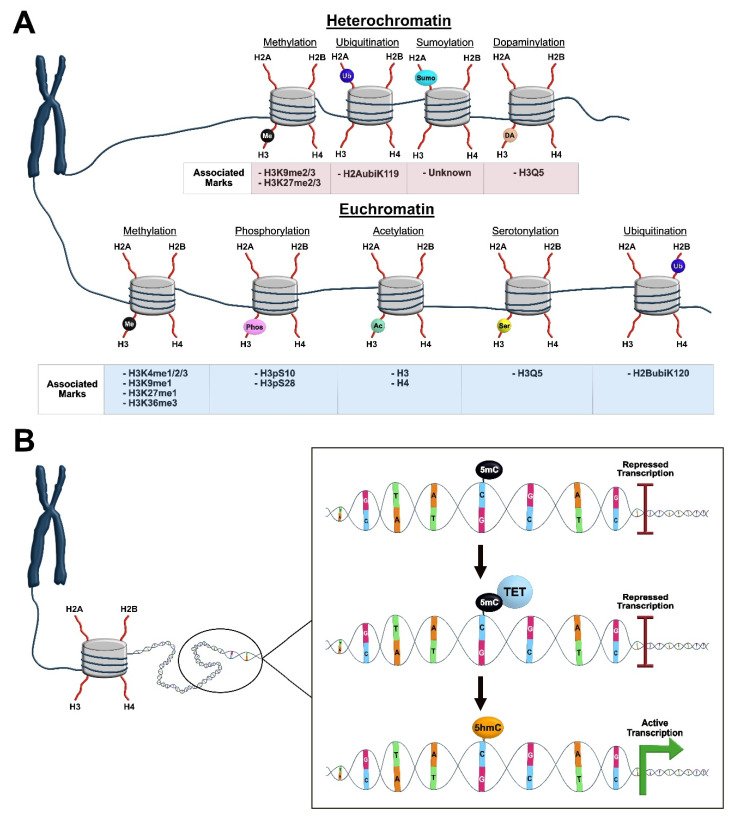
Overview of epigenetic mechanisms. DNA is packaged into the chromosome as chromatin which is wrapped around the nucleosome structure containing two copies each of the core histone proteins (H2A, H2B, H3, and H4) with protruding N-terminal tails. (**A**) Several post-translational modifications (PTMs) can occur at histone proteins to regulate chromatin structure and gene transcription. Epigenetic PTMs of histones associated with a heterochromatin state (top) include methylation (black), ubiquitination (purple), sumoylation (aqua), and dopaminylation (orange). Specific histone modification sites associated with heterochromatin state for each group of PTMs are described as associated marks (red box). Epigenetic PTMs of histones associated with a euchromatin state (bottom) include methylation (black), phosphorylation (pink), acetylation (green), serotonylation (yellow), and ubiquitination (purple). Specific histone modification sites associated with euchromatin state for each group of PTMs are described as associated marks (blue box). (**B**) DNA methylation is an epigenetic mechanism that can be associated with either active or repressed transcription. The circle indicates a portion of DNA that is magnified within the black outlined box (right). The 5mC mark (black) is established on a cytosine residue and is associated with transcriptional repression. The TET enzymes (blue) are recruited to the 5mC mark to initiate the demethylation process. The 5mC mark (black) is converted to the 5hmC mark (orange), which is associated with active gene transcription. Nucleotides are illustrated as follows: Thymine (T; green), Adenine (A; orange), Guanine (G; pink), Cytosine (C; blue).

Epigenetic mechanisms control gene expression through structural changes that make regions of DNA more or less accessible for transcriptional machinery. DNA is tightly packaged into the chromosome through chromatin, which is comprised of long stretches of DNA wrapped around histone proteins, including two copies of each core histone, H2A, H2B, H3 and H4 [7], that are arranged to form the nucleosome. A linker protein, H1, connects the nucleosomes and many nucleosomes form the chromatin material in a cell [8]. Epigenetic mechanisms control changes in chromatin accessibility to either enhance or repress transcription. Specifically, the inactive state of the chromatin (heterochromatin) prevents the transcription of DNA, and the active state (euchromatin) allows transcription [9]. An intermediate state (bivalent chromatin) can also occur, allowing transcription to quickly shift between an active and repressed state. The accessibility of chromatin is controlled by internal changes of the DNA itself or through post-translational modification of the histone proteins within the nucleosome. In the next section, we will focus on DNA methylation and post-translational modification of histone proteins as the epigenetic marks in the normal physiology of the cell. We will also briefly discuss the importance of long non-coding RNAs (lncRNAs) and chromatin remodeling enzymes in epigenetic-mediated transcriptional control. A summary of how some of the more common epigenetic modifications control gene transcription is outlined in Figure 1.

### 2.1. DNA Methylation

DNA methylation is one epigenetic mechanism that causes direct changes to DNA through the addition of a methyl group at cytosine. The enzyme DNA methyltransferase (DNMT) transfers a methyl group from S-adenosyl-methionine (SAM) onto 5’-cytosine positioned adjacent to guanine nucleobases to establish the 5-methylcytosine mark (5mC) [10,11,12,13]. There are different subgroups of DNMTs, which all have unique roles. The de novo DNMTs (3a and 3b) create new methylation marks, while the maintenance DNMT (DNMT1) maintains previously marked methylation on DNA by methylating the opposing DNA strand [14]. Initially, it was shown that DNA methylation represses transcription by blocking transcription factors from binding regulatory sites on DNA [15] and by promoting the heterochromatin state through recruitment of transcriptional repressors [16,17,18]. The establishment of 5mC, by methylation of cytosine residues of DNA, recruits DNA binding proteins containing a methyl-DNA binding domain (MBD) and transcription regulatory domain (TRD). Interestingly, in some cases, extensive methylation was shown to silence a gene completely. However, later studies suggested that methyl-CpG-binding protein 2 (MeCP2), previously thought to only regulate transcriptional repression, could also activate transcription by interacting with the transcription factor CREB [19,20]. Studies also reported functional duality for de novo DNMTs (3a and 3b), whereby they are associated with heterochromatin and euchromatin [21,22]. Importantly, it is now understood that there are different types of DNA methylation marks, which can either activate or repress transcription. Specifically, DNA with the 5mC mark, which is the transcriptionally repressive form, can undergo demethylation via the ten-eleven translocation (TET) enzymes, leading to multiple forms of DNA methylation. During the demethylation process, the TET enzymes oxidize 5mC to 5- hydroxymethylcytosine (5hmC), which can be further oxidized to 5-formylcytosine (5fC), and lastly 5-carboxylcytosine (5caC). Importantly, in post-miotic neurons, the 5hmC mark may be a stable, independent epigenetic mechanism that promotes euchromatin and active transcription, though more commonly is a transient mark as part of the demethylation process [23]. Thus, DNA methylation is a dynamic mechanism employed by the cell to control gene expression.

DNA methylation typically occurs in specific areas, known as CpG sites, where a cytosine residue is followed by a guanine residue. However, DNA methylation may also occur at regions known as non-CpG sites, where the cytosine residues are followed by an adenine, thymine, or another cytosine residue [24,25]. Non-CpG methylation is established by DNMT3A/B and observed genome-wide throughout the brain, although it is less prevalent than CpG methylation. Notably, DNA methylation, especially that which occurs at CpG sites, has been shown to change in response to several experience-dependent events, such as neural activity, estrogen’s effect on human cells, and exercise in muscle [26,27,28,29,30]. Later in this review, we will outline how DNA methylation status is altered during age-related memory decline and Alzheimer’s disease.

### 2.2. Histone Modifications

Post-translational modification of histone proteins is another major epigenetic mechanism responsible for transcriptional regulation. This mechanism of epigenetic tagging can be mediated independent of DNA methylation and is highly important for structural control of chromatin. Histones are highly basic proteins, which use a long stretch of their N-terminal tail to interact with the DNA molecule of chromatin. Structural studies have found that the N-terminal tail of histone proteins protrudes from the chromatin core and is the site of post-translational modifications (PTMs), which are critical regulators of DNA structure and gene expression [31]. When histone proteins are unmodified, their positive charge allows them to interact with the negatively charged DNA, leading to a closed chromatin state, which inhibits gene expression [32]. The N-terminal tail of histone proteins can undergo several covalent modifications, namely acetylation, phosphorylation, methylation, ubiquitination, sumoylation, serotonylation, and dopaminylation, all of which alter the overall chromatin accessibility and binding properties of histone proteins [32,33,34]. These combined PTMs of histone proteins serve as a “histone code” and regulate gene expression by engaging transcriptional machinery [33].

Acetylation, which is arguably the most widely studied PTM of histones, is characterized by the neutralization of positively charged groups of lysine residues by a class of enzymes known as histone acetyltransferases (HATs). HATs function through transferring an acetyl group from acetyl coenzyme A to the lysine residues of the histone tail [35,36,37,38,39]. The acetylation of histones is a reversible process, and the removal of the acetyl group is mediated by enzymes known as histone deacetylases (HDACs). Histone acetylation is generally associated with transcriptional activation through the recruitment of transcription factors and RNA polymerase II, therefore it is considered a mark of euchromatin [40]. CREB binding protein (CBP) is one of the best examples of HAT activity in the regulation of transcription in learning and memory, which will be discussed in more detail in Section 3 [41,42,43,44,45].

Histone methylation is another epigenetic PTM that plays important roles in transcriptional control. The addition of methyl groups to a histone protein is catalyzed by enzymes known as histone methyltransferases (HMTs), which can transfer up to three methyl groups from S-adenosine methionine to the lysine residues of the histone tail [46]. Once the methylation mark is established, it is relatively stable compared to acetylation, which is less stable, and is often involved in long-term maintenance of genes [47,48]. Unlike histone acetylation, histone methylation may lead to transcriptional activation or repression depending upon the methylation pattern. Specifically, the direction of transcriptional control depends on both the number of methyl groups added and which histone site is being modified. For example, trimethylation of histone H3 at lysine 4 (H3K4me3) leads to transcriptional activation, whereas dimethylation of histone H3 at lysine 9 (H3K9me2) is associated with transcriptional repression [49]. Numerous histone methylation marks have been implicated in learning and memory, as discussed in Section 3.

Like histone acetylation, histone phosphorylation is another epigenetic PTM associated with transcriptional activation. Phosphorylation of histone H3 had gained more attention due to its association with the condensation of chromosomes during mitosis [50,51,52]. Interestingly, H3 phosphorylation was first reported in response to the activation of mitogenic signaling pathways [53]. Phosphorylation of histone H3 on the serine 10 residue is mediated by ribosomal protein S6 kinase 2 (RSK2), which is downstream of several other kinases, including extracellular signal-regulated kinase (ERK), mitogen- and stress-activated protein kinase 1 (MSK1), and the aurora kinase family member increase in ploidy 1 (IPL1) [54,55,56]. Recent studies also indicate aurora kinases in H3 serine 28 phosphorylation [57]. Histone phosphorylation is a reversible process during which phosphatases remove phosphate groups from the histones [53,58]. Protein phosphatases 1 (PP1) and 2A (PP2A) have been implicated in the regulation of H3 phosphorylation [56,59]. Taken together, H3 phosphorylation works in concert with other histone modifications to modulate essential cellular functions by regulating transcriptional machinery binding with the chromatin molecule.

Histone ubiquitination, one of the few PTMs in which a protein binds with the histone, is established through the ubiquitin-proteasome system (UPS) that is commonly associated with protein degradation [60]. Briefly, the small regulatory protein, ubiquitin, is activated and transferred to the N-terminal histone tail through a series of ubiquitin-related enzymes. Namely, the ubiquitin ligase E3 enzyme ultimately adds the ubiquitin molecule to the histone tail. Although the main role of the UPS in cells is for protein degradation, not all ubiquitin marks lead to degradation, and histones are typically monoubiquitinated in order to control gene transcription. Like other proteins, histone proteins such as H2A, H2B, and H3 can all be ubiquitinated through the addition of ubiquitin on the amino-terminal of the lysine residue [61,62,63,64] and ubiquitinated histones take part in transcriptional regulation [65,66] as well as many other cellular processes. Histone protein 2A (H2A), which was the first histone identified to be monoubiquitinated in the cell [61], is one of the two most studied forms of histone ubiquitination and is associated with transcriptional repression. The other highly studied form of histone ubiquitination, monoubiquitination of histone H2B at lysine 120 (H2BubiK120), has been shown to be an important epigenetic mark for facilitating transcriptional changes via its regulation of the active H3K4me3 mark [67].

In addition to ubiquitination, histones can also be modified through the addition of the small ubiquitin-like modifier, SUMO. The process of histone sumoylation, similar to that of ubiquitination, requires the coordinated actions of various ubiquitin ligases, which are specific to SUMO and independent of ubiquitin-proteasome signaling. A histone, or any other protein, can only acquire a single SUMO modification, which targets it for a variety of cellular fates that are independent of protein degradation. While protein sumoylation remains one of the less-studied modifications, initial evidence suggests that it is primarily involved in transcriptional repression, and has not been updated to include more diverse functions, including chromatin remodeling [68].

More recently, evidence has emerged that histone proteins can also be modified by monoamines. The first reported neurotransmitter modification of a histone protein was serotonin binding to histone H3 at glutamine 5 (H3Q5), and this process is referred to as serotonylation [69]. This modification was placed by the tissue transglutaminase 2 on histones containing the H3K4me3 mark, which facilitated Transcription Factor II D (TFIID) binding and increased gene transcription [70]. Following this initial report, another study found that H3 could also be modified by dopamine, a process referred to as dopaminylation [71]. This modification again occurred at H3Q5 but, unlike serotonylation, was associated with transcriptional repression. Considering the recent nature of these findings, it is currently unknown whether other neurotransmitters can also modify histone proteins or if these modifications can occur at other histone sites.

### 2.3. Chromatin Remodeling Enzymes

In addition to modification of histone proteins, remodeling of the chromatin structure is another mechanism by which DNA accessibility can be altered to regulate transcription factor binding [72]. This process is regulated by ATP-dependent chromatin remodeling complexes, of which there are four subfamilies: imitation switch (ISWI), chromodomain helicase DNA-binding (CHD), switch/sucrose non-fermentable (SWI/SNF) and INO80. These remodeling complexes all utilize hydrolysis of ATP to disrupt the connection between histones and DNA and, by doing so, are able to regulate histone sliding or ejection as well as incorporation of histone variants. In the case of the latter, H2A variants (H2A.Z, H2A.X) are the most common and, unlike the canonical histone proteins, are poorly conserved across species [73]. These histone variants can have an alternative structure or function from the canonical histone proteins to alter transcriptional regulation and have been implicated in the memory process.

### 2.4. Long Non-Coding RNAs

An expanding category of epigenetic mechanisms includes long non-coding RNAs (lncRNAs), which are defined as transcripts that are greater than 200 nucleotides in length and are not translated into protein [74]. Importantly, lncRNAs regulate gene expression at the DNA level, unlike other types of noncoding RNA, such as microRNAs, which act post-transcriptionally. These non-coding RNAs are capable of binding DNA to regulate transcriptional processes and have been observed to work with other epigenetic modifying enzymes, such as histone methyltransferases. The first evidence implicating lncRNAs in epigenetic-mediated transcriptional regulation came from the process of X-inactivation in females, in which the lncRNA *Xist* plays a major role. Since then, evidence has suggested that lncRNAs can regulate epigenetic processes in numerous ways, including the recruitment of histone modification complexes directly or by acting as a scaffold for histone-modifying enzymes. For example, it has been observed that the lncRNA *Neat1* works with the repressive histone methylation mark, H3K9me2, to regulate gene transcription within the hippocampus [75]. Another lncRNA, *LoNA*, which is nucleolus-specific, has been shown to alter histone methylation status in vitro as well as regulate rRNA production and memory within the hippocampus [76]. The roles of lncRNA in memory, age-related memory decline, and neurodegenerative disorders, such as Alzheimer’s disease, will be discussed in more detail in later sections.

## 3. Inhibitors and Activators of Epigenetic Modifications

Ultimately, the ability to translate data regarding the importance of epigenetic modifications to memory and the treatment of various neurodegenerative disorders will depend on the availability of pharmacological and other genetic tools that can inhibit or activate these processes in the brain. In this section, we will review the literature on broad-spectrum pharmacological manipulations of DNA methylation and histone acetylation, followed by discussing modern genetic tools that can achieve gene- and cell-type specific bidirectional control of various epigenetic modifications in vivo.

### 3.1. Pharmacological Manipulations

With recent advancements in pharmacology, it is now possible to design and screen multiple small molecules which target specific kinases of epigenetic mechanisms such as DNA methylation and histone modifications (acetylation, deacetylation, methylation, and phosphorylation). Currently available DNMT inhibitors (5-AZA and zebularine) are cytosine analogs with similar modes of action [77,78,79]. These compounds are rapidly incorporated into DNA during replication and interfere with the covalent binding of DNMTs with DNA, leading to demethylation and gene reactivation [77,80,81,82,83]. With FDA approval [84], ongoing clinical trials have shown promising results in the treatment of diseases including myelodysplastic syndrome (MDS) and other leukemias [85,86,87] using these drugs.

A number of HAT family members, such as the p300/CBP family and PCAF family [88,89,90], have been identified to regulate gene expression [91,92] through acetylation of histones [88,89,90] or other substrates [93,94]. Several cell-permeable, small molecule modulators with minor homology in sequence and structures have been designed to specifically inhibit HATs [95]. Prior to the specific HAT inhibitors, several cell-impermeable non-specific HAT inhibitors, such as polyamine CoA conjugates [96,97] and natural plant derivatives [98,99] were found to block HAT activity. Despite their application in past studies, many challenges still existed in terms of these drugs’ potency, bio-availability, and cell permeability until recently. A more selective p300/CBP HAT inhibitor known as C646 was used to probe the role of histone acetylation. C646 is a reversible, cell-permeable p300/CBP HAT inhibitor (Ki = 400 nM), which competes with acetyl-CoA for the p300 Lys-CoA binding pocket [100]. The steady-state level of histone acetylation depends on the balance between the activity of HATs that add acetyl groups and HDACs that remove acetyl groups from histone proteins. The HDACs are broadly divided into two classes of isoforms. The class I isoforms include HDACs 1, 2, 3, and 8, while the class II isoforms include HDACs 4, 6, 9, 10, and 11. There are several commonly used HDAC inhibitors (HDI): trichostatin A (TSA) inhibits both class I and class II; sodium butyrate (NAB) and suberoylanilide hydroxamic acid (SAHA) are specific for class I. HDIs block the reversible removal of acetyl groups from the histone tail’s lysine residue, resulting in hyperacetylation of histones and altered gene expression [101,102,103,104,105]. Inhibition of histone phosphorylation is also possible via the specific Aurora kinase B inhibitor AZD1152 [106]. Additionally, there are now numerous inhibitors available for the manipulation of histone methylation mechanisms, including methyltransferases and demethylases, allowing bidirectional control of this histone modification. However, due to the promiscuous nature of histone-modifying enzymes and the gene-specific nature of aberrant epigenetic modifications identified in diseases states, pharmacological approaches have important limitations that can hinder their potential application to the treatment of neurodegenerative disorders.

### 3.2. Genetic Manipulations of Epigenetic Modifications

While pharmacological manipulations of epigenetic modifications have been effective and present some promising therapeutic potential, a rapidly expanding area that can overcome many of the shortcomings associated with this approach is the recent development in genetic tools based on the CRISPR-Cas9 gene-editing system. Notably, numerous catalytically inactive or dead, Cas9 (dCas9) protein fusions have been developed over the last several years, which can control gene-specific epigenetic modifications [107]. For example, it is now possible to create states of DNA 5mc and 5hmc at specific gene promoters using dCas9-DNMT3a and dCas9-TET1 fusions [108,109], respectively, the latter of which has shown promise for the treatment of the neurodevelopmental disorder Fragile X Syndrome [110]. Additionally, various other dCas9-protein fusions have been developed that allow for gene-specific recruitment of histone-modifying enzymes to specific DNA regions, including HATs, HDACs, methyltransferases, and demethylases [111]. Outside of these specific dCas9-protein fusions, alternative platforms can be used to regulate gene expression levels of essentially any epigenetic modifying enzyme. Specifically, the CRISPRa and CRISPRi platforms can be used to broadly upregulate or downregulate gene transcription, respectively, in addition to their ability to achieve cell-type specificity [112]. Furthermore, lncRNAs can be targeted to specific DNA regions using the recently developed CRISPR-Display platform [113]. Thus, while still in its early stages, CRISPR-dCas9 based global and gene-specific epigenetic modifications manipulations may have significant therapeutic potential.

## 4. Epigenetic Code in Synaptic Plasticity and Memory Formation

Some of the first evidence indicating that epigenetic modifications occur in the adult brain came from the process of long-term memory (LTM) formation. In the nearly 20 years since this first study was published, significant evidence has emerged that implicates a wide variety of different epigenetic mechanisms in activity- and learning-dependent synaptic plasticity. In this section, we will discuss how epigenetic mechanisms play a role in synaptic plasticity and memory formation. A summary of the major identified epigenetic modifications in the process of memory formation is summarized in Table 1.

### 4.1. DNA Methylation, Synaptic Plasticity, and Memory Formation

Griffith and Mahler [114] first proposed the role of DNA modification in memory storage. The principle behind this postulation was that DNA acts as an information storage unit upon continuous molecular turnover. Supporting this view, Crick [115] postulated a mechanistic theory of preservation of information in DNA through a maintenance molecule (matching the function of DNMT1) against constant dissipation of acquired changes by molecular turnover. Holliday [116] supported and extended this theory by suggesting that modification of the cytosine residues of DNA provides stability for long-term memory storage. Later on, several studies showed active DNA methylation in several brain regions [117,118,119,120,121] in a time-dependent manner. Recent studies also indicate a cortical layer-specific distribution of DNMTs in the adult human brain [118,122]. The presence of DNMTs in post-mitotic neurons raises the question of their role in the adult brain. To this end, several studies have begun to address this question by investigating the role of DNMTs in learning and memory. Early studies found a change in DNA methylation of genes in the hippocampus upon learning [123,124]. Specifically, upregulation of DNMT gene expression in the hippocampus has been observed following contextual fear conditioning and inhibiting DNMT expression interfered with contextual fear memory formation [121,123,124]. Furthermore, a global inhibition of DNA methylation by DNMT inhibitors modifies methylation of specific memory-related genes including *Reelin, Bdnf* and *Protein phosphatase 1* (*PP1*), and hence alters synaptic plasticity and learning and memory [123,124,125].

Since long-term potentiation (LTP) is thought to be a cellular signature of memory formation, it was expected that epigenetic modifications such as DNA methylation should alter, or be altered by stimuli inducing plasticity. Indeed, altered DNA methylation of the memory enhancing gene *Bdnf* has been observed following synaptic depolarization [126,127]. Since maintenance of remote memory requires separate structures, such as the anterior cingulate cortex [128], studies have also investigated the role of DNA methylation in the maintenance of remote memory [129]. In one study, intracortical infusion of DNMT antagonists 29 days after training blocked memory retention. Observations from this study indicated that altered DNA methylation of memory-inducing (*Reelin* and *Bdnf*) and memory-repressing (*PP1*) gene promoters in the CNS occurs in memory formation and retention. Furthermore, several novel studies have indicated DNMT3A and DNMT3B as demethylating enzymes [26,27] and hence complicated our understanding of DNA methylation in learning and memory. In addition, other researchers have reported the *Gadd45* family as a key regulator of DNA demethylation in the CNS [130,131,132].

In biological systems, it is difficult to establish the incidence of one event independent of others. Likewise, it has been shown that DNA methylation and histone modifications work in parallel to regulate transcription in the formation and storage of memory in the rat hippocampus [133,134,135,136]. The cross-talk between DNA methylation and histone modifications has been clearly demonstrated by a study in which hypermethylation of the *Zif268* gene promoter was correlated with an increase in H3-methylation upon contextual fear conditioning [137]. Taken together, the balance between (1) changes in DNA methylation and (2) the coordinated action of histone modifications may engage several transcription molecules which are currently understood in the context of memory formation and maintenance.

### 4.2. Histone Modifications, Synaptic Plasticity, and Memory Formation

Recently, several studies have indicated a role for post-translational modification (PTM) of histone proteins in synaptic plasticity and memory formation [135,136,138,139,140,141]. Prior to mammalian studies, several groups used *Aplysia* and crab models to elucidate the role of histone acetylation in memory formation. The *Aplysia* model has been used to demonstrate the role of serotonin (5-HT) in memory formation by facilitating synaptic responses [142]. It was later shown that 5-HT also induces acetylation of H3 and H4 proteins at the C/EBP promoter region [143]. Inhibition of HDACs by TSA causes long-term facilitation (LTF) with just 1 pulse of 5-HT, which proves that 5-HT induces LTF by regulating histone acetylation or deacetylation activity. In another study using the crab model, Federman et al. demonstrated that strong training in the context-signal memory paradigm enhances LTM formation by inducing H3 acetylation [144]. Interestingly, inhibition of HDACs by TSA also causes the formation of LTM when using a weak training protocol. Taken together, these studies suggest a role for histone acetylation and deacetylation in memory formation in invertebrates.

Using other experimental paradigms, studies have focused on histone acetylation and deacetylation in LTP modulation in the mammalian hippocampus as a means of understanding memory formation. One study found that induction and maintenance of late LTP (L-LTP) by HDAC inhibition is transcription-dependent [138]. In another study, pairing a sub-threshold stimulus with an HDAC inhibitor induces a PKA/CREB transcription-dependent L-LTP in the hippocampal CA1 region [45]. The CREB-binding protein (CBP) has intrinsic HAT activity, and it has been found that *Cbp* +/− mice are L-LTP deficient [42]. Interestingly, HDAC inhibition was able to restore L-LTP, which indicates that the reduced L-LTP in those mice is due to a deficiency of HAT activity. In addition, it is also reported that the application of TSA enhanced forskolin-induced LTP in amygdalar slices. Since LTP is thought to be a cellular mechanism of memory formation, these studies show that histone acetylation and deacetylation play a major role in hippocampal and amygdalar synaptic plasticity, as well as memory formation.

Behaviorally, contextual fear conditioning in rodents has served as a model to study LTM formation in mammals. It has been found that contextual fear conditioning in rodents is associated with a transient increase of H3 acetylation, but H4 acetylation remains unchanged, though the latter has been shown to increase following training on a non-aversive spatial task [138,145]. In addition, injection of an HDAC inhibitor 1 h before contextual fear conditioning caused increased freezing behavior when assessed 24 hrs after the test, suggesting long-term fear-enhanced memory formation. CBP, with its intrinsic HAT activity, recruits many other transcriptional co-activators to induce gene transcription, and it has been indicated that heterozygous mutation of *Cbp* causes cognitive disorders, including Rubinstein-Taybi syndrome, characterized by severe mental retardation [146]. Considerable advances in genetic engineering allow us to alter specific genes of interest. Using this, Korzus et al. have generated transgenic mice carrying a dominant-negative *Cbp* transgene which specifically blocks HAT activity with an inducible tet system [43]. These mice were deficient in declarative and spatial memory formation, while contextual fear memory was intact. The behavioral phenotype was reversible upon turning off the transgene. Similarly, Alarcon et al. used CBP +/− heterozygous mice to assess the role of CBP HAT activity in memory formation [42]. They have found that CBP +/− heterozygous mice froze less than control animals in the contextual fear conditioning test, but showed no difference in latency and path length in the Morris water maze (MWM) spatial memory test. Administration of the HDAC inhibitor restored the deficit LTM in both transgenic and mutant mice. Besides CBP, two other transcriptional co-activators, p300 and p300/CBP associated factor (PCAF), also have acetyltransferase activity and play roles in LTM formation [41,147]. These studies underpin the importance of CBP and other transcription co-activators with HAT activity in gene transcription in memory formation.

Many other studies also examine the role of histone acetylation in memory formation. Training for eye-blink conditioning and object recognition memory induces H3 acetylation, and inhibition of HDAC causes enhanced memory formation with this training [148]. Several studies have found an increase in histone acetylation in the BDNF promoter region in the hippocampus and the prefrontal cortex upon a fear conditioning test [124,149]. More recently, it was shown that a weak training stimulus that is unable to form LTM, when paired with HDAC inhibitor, induces LTM formation [150]. This is in line with the observation of Vecsey et al., who reported that a single train of high-frequency stimuli, that normally generates E-LTP, can induce transcription-dependent L-LTP when paired with HDAC inhibitors [45]. Protein phosphatase 1 (PP1) acts as a memory suppressor gene and inhibition of PP1 has been shown to induce acetylation of H2B, H3, and H4 to promote LTM formation in the MWM task and object recognition test [151]. Thus, this study underlines a mechanistic way in which histone acetylation by PP1 could support LTM formation. Considering the cross talk between DNA methylation and histone acetylation, it has been found that inhibition of DNMTs blocks training-induced H3 acetylation, which could be rescued with HDAC inhibition [152], pointing to a complex interaction between these two mechanisms in memory formation. These studies also suggest that HDAC might act as a negative constraint on memory formation [153]. Indeed, accumulating evidence supports this by showing that overexpression of the HDAC2 gene impaired, but deficiency of HDAC2 enhanced LTP as well as memory formation [154].

Histone phosphorylation is another PTM that provides a unique epigenetic mark to regulate chromatin dynamics [136]. In this regard, the mitogen-and stress-activated protein kinase 1 (MSK1) plays a major role in bringing on the function of histone phosphorylation. Consistent with this, germline knockout of MSK1 impairs long-term spatial and contextual fear memory formation, leaving cued fear memory intact [155]. In contrast to the previous findings, HDAC inhibitors failed to rescue the memory deficit in MSK1 knockout mice, suggesting a critical interrelation between histone acetylation and phosphorylation through a common upstream regulator of both. In addition to MSK, another kinase complex known as the IκB kinase (IKK) complex also regulates histone phosphorylation in the hippocampus [134]. Taken together, these studies indicate a critical role of histone kinases in memory formation.

Over the last decade, strong evidence has implicated transcriptionally active and repressive histone methylation in memory formation. Early evidence indicated that H3K4me3 was increased in the hippocampus of rodents that underwent contextual fear conditioning and that genetic deletion of its methyltransferase, *Mll1*, impaired long-term memory [137]. This evidence was supported by subsequent studies [156,157], strongly implicating a role for H3K4me3 in transcriptional regulation necessary for LTM formation and storage. Interestingly, several studies have shown a role for transcriptionally repressive H3K9me2 and H3 lysine 27 trimethylation (H3K27me3) in memory formation and stability in the hippocampus [158,159,160], suggesting that both active and repressive histone methylation is required for LTM formation. Outside of the hippocampus, H3K9me2-mediated transcriptional repression has been shown to be important for fear memory formation in the entorhinal cortex and amygdala [158,161].

While less studied, recent evidence from our group suggests a role for histone ubiquitination in memory formation [162]. We found that learning in a contextual fear conditioning paradigm increased global and gene-specific H2BubiK120 levels in the rat hippocampus. Genetic loss of H2BubiK120 caused a reciprocal reduction in H3K4me3 at target genes, which was regulated by the proteasome subunit RPT6. Consistent with this, loss of H2BubiK120 impaired LTP and memory formation, the latter of which could not be rescued by upregulation of H3K4me3, indicating that without H2BubiK120, the H3K4me3 mark could not be correctly targeted to DNA regions. These data strongly suggest that H2BubiK120 regulates histone “cross-talk” mechanisms critical for memory formation.

In addition to PTMs, the canonical histones can be substituted with variants that have a dynamic impact on downstream transcriptional processes. In the context of memory formation and storage, the only two histone variants studied to date are H2A.Z and H2A.X. In response to fear conditioning, H2A.Z is actively exchanged in the hippocampus and cortex, serving as a negative regulator of the “systems consolidation” process [163]. This learning-dependent exchange of H2A.Z is associated with transcriptional repression, and it has been demonstrated that viral-mediated depletion of H2A.Z leads to enhanced fear memory and gene transcription [164]. Furthermore, pharmacological inhibition of TIP60, which is part of the H2A.Z deposition complex, in the hippocampus 23 h after learning impairs remote, but not recent, contextual fear memory [165]. Surprisingly, an H2A.Z conditional knockout (cKO) enhanced fear memory in male, but not female, mice [166]. Conversely, cKO of H2A.Z enhanced memory for a non-aversive spatial task in both sexes, while stress-enhanced fear learning was reduced to a greater extent in females. These data strongly suggest a sex- and task-specific role for H2A.Z in memory formation. While fewer studies have examined H2A.X in the brain, increasing evidence suggests a role for this mark in synaptic plasticity and memory formation. Phosphorylation of H2A.X at serine-139 is associated with double-strand breaks (DSBs) in DNA. Interestingly, recent evidence indicates that DNA DSBs occur in hippocampal cultures in response to cellular stimulation and in the intact hippocampus following contextual fear conditioning training and memory retrieval [167,168,169]. Additionally, siRNA-mediated knockdown of *TopIIβ*, which causes DSBs in DNA, prevents the retrieval-induced increase in H2A.X phosphorylation and impairs contextual fear memory following retrieval. Collectively, these data strongly implicate a role for H2A variants in synaptic plasticity and memory formation in the brain.

### 4.3. Chromatin Remodeling Enzymes and lncRNAs in Synaptic Plasticity and Memory Formation

Several studies have implicated a role for chromatin remodeling enzymes and lncRNAs in synaptic plasticity and/or memory formation. Genetic manipulation of the highly conserved chromatin assembly and remodeling factor, *Chd1*, alters the expression of several memory-permissive genes and impairs LTM [170]. Additionally, several studies have shown that the nBAF complex, which belongs to the SWI/SNF subfamily, is critically involved in memory formation and synaptic plasticity [171,172]. Furthermore, brain-specific deletion of the chromatin remodeling enzyme, *Atrx*, impairs aversive and non-aversive spatial memory [173]. Knockdown of the lncRNA *Neat1* enhances hippocampus-dependent memory in mice, which likely occurs due to its regulation of H3K9me2 [75]. Expression of *Lym-NOS1AS*, a novel molluscan nitric oxide synthase-related lncRNA, correlates with memory performance after the training, suggesting its role in memory formation [174]. Furthermore, RNA sequencing analysis of prefrontal cortex tissue from fear conditioned mice revealed downregulation of *Gomafu* and knockdown of this lncRNA induced anxiety-like behavior [175]. Lastly, when the lncRNA *LoNA* was knocked down in the hippocampus of mice, several synaptic proteins were elevated, and animals had better performance in the Morris water maze compared to control mice. Interestingly, overexpression of *LoNA* in the hippocampus led to impaired spatial memory [76]. Collectively, these studies reveal a potential role for chromatin remodeling complexes and lncRNAs in synaptic plasticity and memory formation, though much still remains unknown, especially for the latter, where we are only beginning to understand their function(s) in the brain.

## 5. Epigenetics in Age-Related Memory Decline

Over the last decade, a number of studies have begun to explore how the brain epigenome changes across the lifespan and contributes to age-related memory decline, a significant risk factor for the development of Alzheimer’s disease and related dementias. As an excellent recent review has been published on this topic [5], we will briefly summarize some of the significant findings from the last decade.

Numerous studies have found alterations in DNA methylation that are associated with age-related memory loss. Repressive DNA 5mc has been reported to be increased at plasticity-related genes in the prefrontal cortex of aged rodents, which was associated with gene repression [176]. Additionally, the memory permissive genes *Arc* and *Egr1* have altered resting levels of DNA methylation in the aged hippocampus, which becomes further altered following learning [177,178]. These changes in DNA methylation are associated with altered expression of DNA methylation enzymes, including reductions in DNMT1 and DNMT3a expression in the aged hippocampus [179]. Interestingly, upregulation of *Dnmt3a* or *Tet2* expression can actually rescue age-related memory impairments [180,181], emphasizing the importance of altered DNA 5mc and 5hmc levels in age-associated memory loss.

Several histone modifications have also been shown to become dysregulated in the brain across the lifespan and be associated with age-related memory loss. The aged brain has lower levels of histone acetylation [182], which is associated with higher activity of HDACs. Pharmacological and genetic repression of HDACs enhance histone acetylation levels and rescue age-related memory loss [183,184]. While the mechanisms by which increased HDAC activity leads to memory decline during aging remain largely unknown, some evidence suggests that it could be due to targeting of *Nr4a* and *Per1* [185,186], two genes critically involved in memory formation. Histone methylation may also contribute to age-related memory loss, though the evidence for this modification remains limited. For example, pharmacological inhibition of H3 lysine 9 trimethylation (H3K9me3), a repressive mark usually associated with genomic imprinting, in the hippocampus improved spatial memory in aged rodents [187]. In the hippocampus, aberrant learning-related changes in H3K4me3 and H3K9me2 have been observed in aged animals relative to young ones [188]. Interestingly, altered H3K9me2 levels in the aged brain may be due to expression changes in *Neat1*, which accumulates in the hippocampus across the lifespan [75]. Outside of acetylation and methylation, no other histone PTM has been implicated in age-related memory loss, though considering their interactive nature, it is likely that other modifications are altered as well in the aging brain.

Recently, evidence has emerged that the levels of histone variants change in the brain across the lifespan and may be involved in age-related memory decline. Both H2A.Z and H3.3 accumulate in the brain with age [164,189]. Despite this, in the hippocampus of young and old animals, learning-induced removal of H2A.Z, which was associated with increased gene expression. Interestingly, even though H2A.Z removal was a common characteristic of learning across the lifespan, the genes this occurred at were distinct in young vs. old mice. Together, these data suggest that changes in the gene-specific targeting of histone variants may contribute to changes in memory across the lifespan.

## 6. Epigenetics in Alzheimer’s Disease-Related Memory Loss

**Figure 2:**
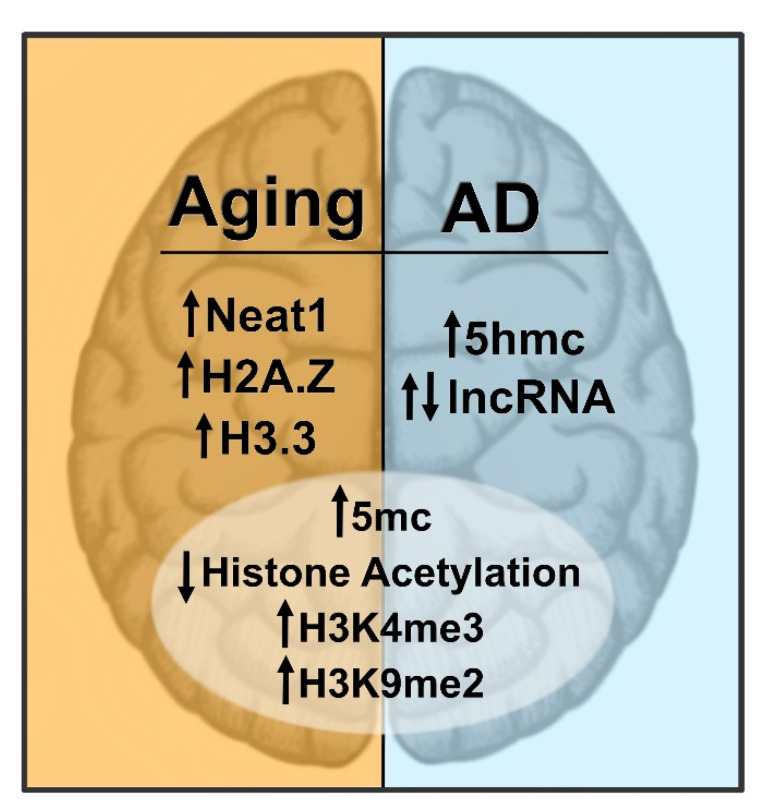
**Epigenetic marks altered in the brain during Alzheimer’s disease (AD) compared to normal aging.** This figure compares several epigenetic marks dysregulated in the brain of AD patients and rodent models with that of normal aging. The marks that change similarly in both conditions are in the circle and marks that change only in one condition are listed separately. The arrows show the direction of the marks’ changes.

In addition to the normal aging process, dysregulation of epigenetic mechanisms has also been widely implicated in the pathophysiology underlying numerous neurodegenerative disorders, many of which are associated with memory loss. In this section, we will discuss epigenetic changes associated with the most prevalent neurodegenerative disorder, Alzheimer’s disease (AD). Important here is that the focus is on how alterations in epigenetic modifications may contribute to memory loss associated with AD, as opposed to disease progression itself. For a more detailed discussion of epigenetic changes in neurodegenerative disease progression, we refer the reader to some excellent reviews published on this topic [6,190]. Important is the comparison between epigenetic dysregulation during normal aging-associated and AD-related memory loss, which is summarized in Figure 2.

AD is the leading cause of dementia, affecting more than 10% of the population over the age of 70. This disorder is characterized by an abnormal accumulation of tau protein in the brain, with the earliest development occurring in regions preferentially involved in memory formation and storage [191]. Numerous studies have also implicated epigenetic dysregulation that is associated with memory loss from AD. For example, histone acetylation levels are reduced in the hippocampus of *APP*/*PS1* mice following fear conditioning, which may be due to elevated levels of HDAC activity. Consistent with this, contextual fear conditioning and LTP deficits present in humanized *APP*/*PS1* mice can be rescued via HDAC inhibition [192,193,194]. Furthermore, in mouse models of AD, H3K9me2 levels are elevated in the prefrontal cortex, and inhibition of the methyltransferases for this mark rescued deficits in recognition memory, working memory, and spatial memory [195]. Similarly, H3K4me3 levels are increased in the prefrontal cortex in AD mouse models, and pharmacological inhibition of this mark leads to recovery of synaptic function and memory impairments [196]. Increases in DNA 5mC and 5hmC levels have been reported in the hippocampus of AD patients [197], though 5mC was decreased in the hippocampus of a mouse AD model with no changes in 5hmc [198]. However, the liver X receptor agonist, GW3965, which improves cognition in mouse models of AD, is associated with altered DNA methylation at a number of plasticity-related genes in the hippocampus, including those involved in the synaptic structure and neurogenesis [199], suggesting a potential role for changes in hippocampal DNA methylation in AD-associated memory loss. Several studies have also observed aberrant DNA methylation profiles in numerous brain regions, including at both CpG and non-CpG sites, indicating altered DNA methylation may be involved in neurodegeneration [24,25]. Evidence from human studies has also widely investigated the correlation between DNA methylation and AD-related genes in post-mortem brain tissues. There are data indicating a hypomethylation in *PSEN1* and *PSEN2*, key enzymes involved in generating amyloid-β peptides, both in CpG and non-CpG sites of AD patients brain tissues [200,201,202]. However, there is no consensus in the literature on whether the methylation of *APP*, the amyloid precursor protein, is significantly altered in AD patients’ brain [203,204,205]. Nevertheless, other AD-associated genes have been found to be differentially methylated in AD patients, such as *APOE* [206], *PLD3* [207,208], and *EPHA1* [201].

Additionally, hundreds of lncRNAs have been shown to have altered expression in the hippocampus of AD mouse models [209]. Consistent with this, treatment of *APP*/*PS1* mice with Danggui-Shaoyao-San, which improves cognitive functioning with age, leads to differential expression of 285 lncRNAs in the hippocampus [210]. Additionally, one study found increased levels of the lncRNA *LoNA* in the brain of *APP*/*PS1* mice and observed rescued learning and memory deficits following *LoNA* knockdown in the hippocampus of *APP*/*PS1* mice compared to controls [76]. However, to date, whether manipulation of specific lncRNAs can rescue AD-associated memory loss has yet to be explored in detail. While several marks have yet to be explored, together, these data do show that wide-scale dysregulation of epigenetic modifications may contribute to AD-related memory impairments.

## 7. Future Directions and Conclusions

Epigenetic mechanisms are diverse and new modifications continue to be discovered. In the last two decades, strong evidence has emerged that these epigenetic mechanisms are critically involved in memory formation in the brain. However, despite the abundance of modifications that have been studied and implicated in the process of memory formation, much still remains unknown. For example, a number of histone modifications have yet to be examined in the context of memory, including histone sumoylation and various forms of histone methylation. In addition, we have only begun to examine the role of histone variants in learning and memory, with the focus thus far primarily being on H2A.Z, and few studies have examined the importance of diverse lncRNAs and chromatin remodeling complexes. Furthermore, much less is known about how these mechanisms become dysregulated across the lifespan and contribute to age-related and AD-associated memory loss. In the context of normal memory, few studies have examined other stages of memory storage with, to date, only a handful of papers on the epigenetic mechanisms involved in the post-retrieval reconsolidation or extinction of fear-based memories [149,157,211,212], though the reasons for this are largely based on technical limitations, especially in terms of reconsolidation. Regardless, a better understanding of how the same epigenetic modifications contribute to different stages of memory storage, especially retrieval, will be critical for fully understanding the cognitive decline associated with age and AD.

## Figures and Tables

**Table 1:** List of known epigenetic marks altered during memory formation in a healthy brain.

The Epigenetic Mark	Brain Region	Direction
5mC	Hippocampus	Up (121)
5hmC	Hippocampus and Anterior Cingulate Cortex	Up (155)
H2BubiK120	Hippocampus	Up (160)
H2B Acetylation	Hippocampus	Up (149)
H2A.Z	Hippocampus and Cortex	Down (161)
H2A.X	Hippocampus	Up (165–167)
H3K4me3	Hippocampus	Up (135, 154, 155)
	Amygdala	No Change (159)
H3K9me2	Hippocampus, Entorhinal and Amygdala	Up (156, 159)
H3K27me3	Hippocampus	Up (157, 158)
H3 Acetylation	PFC, Hippocampus, Amygdala	Up (136, 146, 147, 149)
H4 Acetylation	Hippocampus	No Change (136, 147)
		Up (143)

## Data Availability

All data is included within the manuscript text.
